# Broadband Ultrathin Transmission Quarter Waveplate with Rectangular Hole Array Based on Plasmonic Resonances

**DOI:** 10.1186/s11671-019-3200-y

**Published:** 2019-12-18

**Authors:** Yu Wang, Yumin Liu, Jing Li, Chang Liu, Zhongyuan Yu, Han Ye, Li Yu

**Affiliations:** State Key Laboratory of Information Photonics and Optical Communications, Beijing University of Posts and Telecommunications, Beijing, 100876 China

**Keywords:** Polarization, Metasurface, Quarter waveplate, Communication band, Transmission

## Abstract

The control of the polarization states of light plays an important role in modern optical systems. However, traditional polarization manipulating devices often have narrow bandwidth and their large size makes it difficult for them to achieve miniaturization and integration of optical systems. This work presents an ultrathin quarter waveplate with a periodic silver film 2 × 2 rectangular hole array with a thickness less than λ/50. Numerical simulation shows that the waveplate can efficiently transform a circular polarized wave into a linearly polarized one at the center of 1550 nm, and its bandwidth is 525 nm. Furthermore, the quarter waveplate can efficiently invert linear polarization into circular polarization at 1550 nm, which ellipticity is near unit. With an array of small holes on a metal film to enhance transmission, this structure can increase the transmission to 0.44. The broadband quarter waveplate can be used in communication system and near infrared band system, and be integrated with other optical devices at nanoscale to achieve polarization operation, detection, and sensing.

## Introduction

There is an increasing interest in manipulating the polarization of light in a variety of optical applications, such as polarizers, waveplates, and lenses. Among these, waveplates are important photonic components because it can introduce a specific phase difference, such as π/2 and π, to produce different polarized light to achieve a quarter or half waveplate. Traditional waveplate design uses birefringence of crystals to impose different phases on the incident light. However, the birefringence effect is very weak in natural crystals, resulting in waveplates having a thickness of several hundred microns. Bulky optical components often suffer from difficulties in integration and depth of phase modulation [1–4]. In recent years, the emergence of nanophotonics has opened up a new direction for studying the interaction between light and matters. Especially, nanophotonic devices (thickness about tens of nanometers) can break through the diffraction limit without electromagnetic interference. It has great potential in replacing the large-scaled devices. Among them, nanophotonic devices based on metasurface have attracted more and more attention. The development of metasurface theory and fabrication technology makes it possible to develop nanodevices [5].

Metasurfaces are planar structures that locally modify the polarization, phase, and amplitude of light in reflection or transmission, thus enabling lithographically patterned flat optical components with functionalities controlled by design. It usually have a thickness less than the wavelength. In the process of transmission or reflection, anisotropic metasurfaces produce different phase and amplitude corresponding to TE and TM waves, which provides great flexibility for the design of functional metasurfaces. We can use this to design such as lenses, phase-plates, waveplates, polarizers, beam-splitters, arbitrary vector beam generators and so on [6–17].

Metasurface quarter waveplates based on plasmon resonances are one of the hotspots in recent years [18–24], and timeline published literatures indicate a continuous progress in this area. In 2011, Zhao et al. designed and studied the performance of orthogonal elongated silver nanorod array as a broadband quarter waveplate. It can introduce a 90° phase shift over a thickness of 60 nm [25]. Inspired by the Babinet’s principle, in 2013, the same group designed a quarter waveplate of nanoslits, and achieved circular-to-linear (CTL) polarization conversion in the visible light region. The thickness of the metal layer is reduced to 40 nm [26]. The above two designs have a wide band from CTL polarization. However, it is difficult to achieve the same amplitude of two orthogonally polarized beams. Soon after the pioneering work of Zhao et al., in 2012, Roberts et al. proposed a quarter waveplate with a periodic array of cross-shaped apertures in a silver film. The transmission efficiency and phase (for fixed arm width) of the waveplate are sensitive to the length of the related arm. The conversion from linear-to-circular (LTC) polarization is achieved at some discrete wavelengths from 710 to 760 nm, and the thickness of silver film is 140 nm [27]. It can well achieve LTC polarization, but the wavelength is fixed at specific wavelengths only, and the metal layer is relatively thick. Similarly, based on the anisotropy caused by the arm length in the orthogonal directions, in 2013, Yang et al. proposed a quarter waveplate consisting of a periodic planar array of symmetrical L-shaped plasma antennas. The ellipticity of transmitted light can reach 0.994 at 1550 nm. The bandwidth with ellipticity greater than 0.9 is 80 nm [28]. The waveplate’s circular polarizability nearly unit, yet its bandwidth, is not ideal. By carefully designing the nanoantennas in the superuints, in 2015, Li et al. achieved a quarter waveplate consisting of a 20-nm-thick gold nanorod array. It can theoretical realize the conversion from CTL polarization and reverse transformation around 1550 nm. The circular polarizability is 0.67, and the transmission efficiency is 0.4 [29]. The ultrathin structure can realize CTL polarization in a wide band, but the ellipticity (amplitude ratio) of LTC polarization at 1550 nm is low. Furthermore, in 2017, Zhu et al. proposed a broken rectangular annulus array quarter waveplate. It is formed by two pairs of slits with perpendicular orientation embedded in a 10-nm-thick silver film. It has a 120 nm CTL polarization bandwidth. Also, the waveplate can achieve LTC transformation with the circular polarizability of 0.97, and the transmission efficiency is 0.4 at 1550 nm [30]. It achieves high polarization conversion at the expense of bandwidth.

Through the above examples, generally, as an ideal miniaturized transmission quarter waveplate used in communication band, it should have the following characteristics: firstly, it can realize the conversion from CTL polarization (LTC polarization) in wide band. Secondly, it can achieve the circular polarizability near unit at 1550 nm. Thirdly, the overall transmittance should be as high as possible (the maximum transmittance of an ultrathin quarter waveplate without loss must be 0.5 calculated by the surface admittance theory). Fourth, it should be ultrathin and cost effective. But for now, most of them are still theoretical design, and few experiments have been carried out. Because the ratio of height to width is too high, or the structural parameters are too sensitive to errors, etc., it will affect the performance of the actual quarter waveplates.

Based on the four characteristics above, we propose a transmission quarter waveplate used in communication band. The unit cell is composed of 27-nm-thick holed silver film and silica substrate. Four-hole design avoids the disadvantage of narrow bandwidth of single resonator. They can enhance the localized surface plasmons, thereby increasing the phase anisotropy to introduce abrupt phase shifts, and largely reducing the thickness of the metal layer. Furthermore, the waveplate can achieve 90° phase difference in 525 nm bandwidth. Especially, the circular polarizability is near unit with the transmission efficiency of 0.44 at 1550 nm.

## Methods

**Fig. 1:**
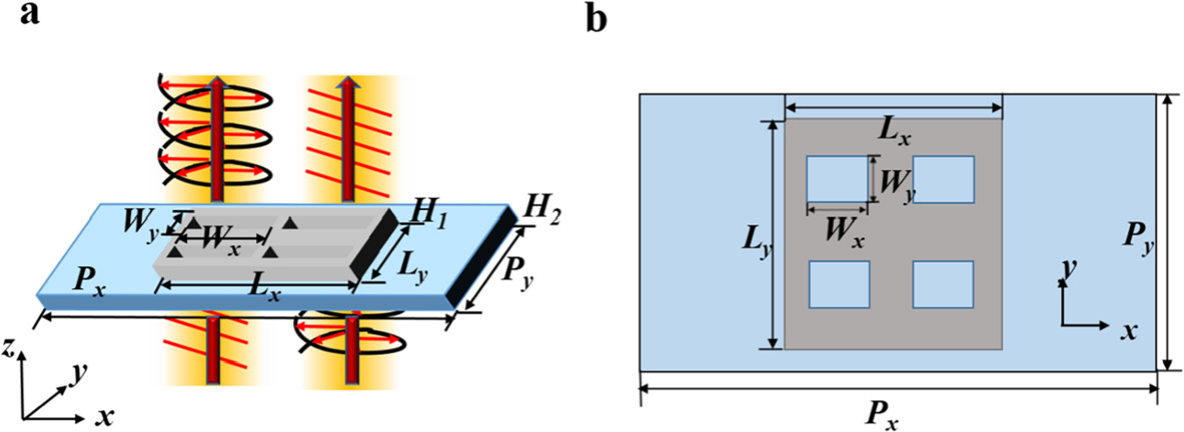
Schematics of the quarter waveplate. The lights are normally incident from the bottom. **a** 3D view of the quarter waveplate. **b** Top view of a unit structure

Figure 1 schematically depicts a unit cell of the proposed plasmonic quarter waveplate, a hole-digging silver film placed on a silica substrate. Four rectangular apertures are arranged in two rows and two columns. The waveplate immersed in an environment of air with refractive index *n* = 1. The silica is assumed to be nondispersive (${\varepsilon}_{SiO_2}=1.47$), and the permittivity of silver is described by Drude model [25]:

1 $$ {\varepsilon}_{Ag}={\varepsilon}_0\left[{\varepsilon}_{\infty }-\frac{f_p^2}{f\left(f- i\gamma \right)}\right] $$

where *ε*_∞_=5, *f*_*p*_ = 2.175 PHz, and *γ* = 4.35THz. The thickness of the silica substrate and silver film is fixed at *H*_*1*_ = 30 nm and *H*_*2*_ = 27 nm, the period of the unit is *P*_*x*_ = 1200 nm and *P*_*y*_ = 500 nm, the length and width of the silver film are *L*_*x*_ = 450 nm and *L*_*y*_ = 480 nm, respectively. The internal dimensions of the apertures *W*_*y*_ = 80 nm is kept fixed, and the length *W*_*x*_ is variable. The center of the apertures are *x* = ±75 nm, *y* = ±110 nm. The numerical simulation is performed by three-dimensional finite-difference time-domain (FDTD) methods, in which the periodic conditions are applied in the *x-* and *y-*directions, and perfectly match layers are used along *z-*direction to make sure that the complete absorption of the excitation light without reflection. Plane waves are normally incident from the underneath of the substrate within the wavelength region from 1000 to 2000 nm. *T* is the normalized total transmittance, and the transmittance in *x-* and *y*-directions is *T*_*x*_ and *T*_*y*_, respectively. We first consider the transmission characteristics of an ultrathin planar metasurface with subwavelength thickness *d* ≪ *λ*_0_ placed on the plane *z* = 0. The transmission can be simply expressed using the Jones matrix:

2 $$ \boldsymbol{T}=\left(\begin{array}{cc}{T}_{xx}& {T}_{xy}\\ {}{T}_{yx}& {T}_{yy}\end{array}\right) $$

where *T*_*ij*_ represents the complex amplitude of the transmitted wave, linearly polarized in the *i* direction for excitation in the *j* direction. Thus, *T*_*xx*_ and *T*_*yy*_ are the copolarization transmission coefficients, and the *T*_*xy*_ and *T*_*yx*_ are the cross-polarization transmission coefficients. Consider the incoming plane wave propagates along the *+z*-direction, the electric field can be expressed as:

3 $$ {\boldsymbol{E}}_{in}\left(\boldsymbol{r},t\right)=\left(\begin{array}{c}{I}_x\\ {}{I}_y\end{array}\right){e}^{i\left( kz-\omega t\right)} $$

where *ω* represents frequency, *k* is the wave vector, and *I*_*x*_, *I*_*y*_ are the complex amplitudes. The matrix *I* =$$ \left(\begin{array}{c}{I}_x\\ {}{I}_y\end{array}\right) $$ determines the state of polarization and the total intensity of the wave. When the linearly polarized light is incident normally at a 45° polarization angle to the *x-*axis, ∣ *I*_*x*_∣ = |*I*_y_∣ = $\frac{1}{\sqrt{2}}$. The transmitted electric field can be described as:

4 $$ {\boldsymbol{E}}_t\left(\boldsymbol{r},t\right)=\left(\begin{array}{c}{T}_x\\ {}{T}_y\end{array}\right){e}^{i\left( kz-\omega t\right)} $$

The incident and transmission fields are correlated by Jones matrix: ***E***_*t*_ *= T****E***_*in*_, that is

5 $$ \left(\begin{array}{c}{T}_x\\ {}{T}_y\end{array}\right)=\left(\begin{array}{cc}{T}_{xx}& {T}_{xy}\\ {}{T}_{yx}& {T}_{yy}\end{array}\right)\left(\begin{array}{c}{I}_x\\ {}{I}_y\end{array}\right) $$

For a medium that has no linear polarization conversion effect (*T*_*xy*_ and *T*_*yx*_ equal to zero [25, 27]), the transmitted field can be expressed as [16]:

6 $$ \left(\begin{array}{c}{T}_x\\ {}{T}_y\end{array}\right)=\left(\begin{array}{c}{T}_{xx}{I}_x\\ {}{T}_{yy}{I}_y\end{array}\right) $$

The phase difference is *△φ = φ*_*y*_ *- φ*_*x*_ between the transmission coefficients *T*_*xx*_ and *T*_*yy*_. For a quarter waveplate, the *△φ* needs to be equal to *(2 m + 1)π/2*, where *m* is an integer.

## Results and Discussions

**Fig. 2:**
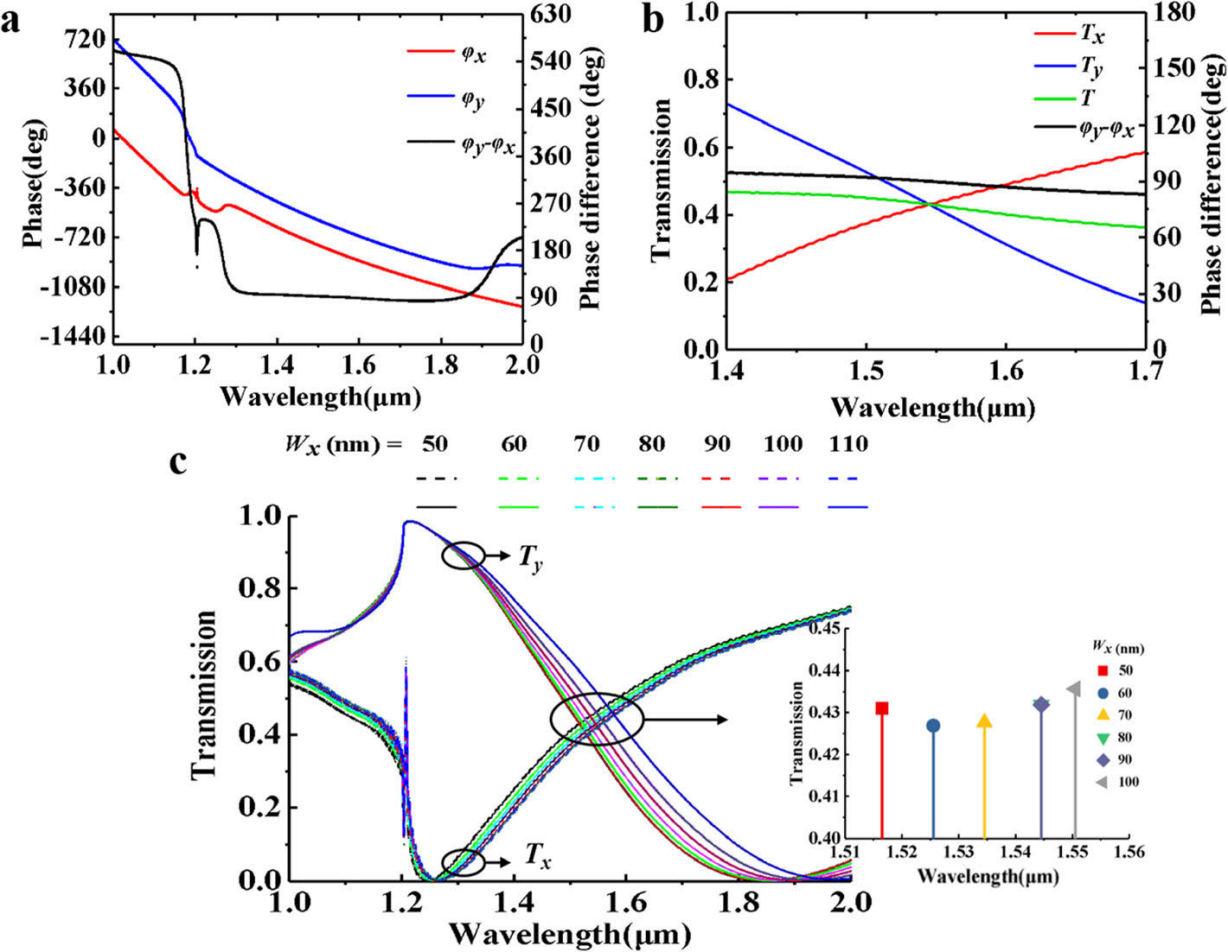
Simulation results of the proposed structure. **a** The phase of *T*_*x*_, *T*_*y*_ and there difference when *W*_*x*_ = 100 nm. **b** Transmittance *T*, *T*_*x*_ and *T*_*y*_, and the phase difference of the two transmission lights. **c** *T*_*x*_ and *T*_*y*_ curves when *W*_*x*_ changes. The small image is the detailed diagram near 1550 nm. It shows the variation trends of *T*_*x*_, *T*_*y*_, total transmittance *T*, and phase difference at the communication wavelength

Simulated phase shifts *φ*_*x*_, *φ*_*y*_ and there difference are shown in Fig. 2a. The *△φ* drops sharply at 1200 nm and eventually stabilizes around *△φ* = 90°. The transmittance curves and phase difference near 1550 nm are shown in Fig. 2b. Generally, a quarter waveplate with a phase difference of 90° ± 5° can be regarded as working normally. For 1328 nm, the *△φ* = 95°, and for 1853 nm, *△φ* = 85°, that means within the near infrared bandwidth of 525 nm, our design can realize the conversion from circular polarization to linear polarization. This is excellent in the current published bandwidth of near infrared quarter waveplate.

The changed size *W*_*x*_ of the hole has different effects on *x-* and *y-*polarization. Figure 2c depicts the transmittance when *W*_*x*_ changes. The peak of *T*_*y*_ and the very sharp peak of *T*_*x*_ at 1200 nm are related to the *P*_*x*_ = 1200 nm. The condition for Wood’s anomalies to occur is *λ = p(*sin*θ*_*i*_ *+ 1)* [31, 32] and *θ*_*i*_ is 0 for normally incident wave; therefore, the peak occurs when *λ = P*_*x*_. Also, with the decrease of *P*_*y*_, the valley of *T*_*x*_ shifts to the short wavelength direction, and *T*_*y*_ moves towards long wavelength direction, resulting in the change of wavelength and transmittance corresponding to the intersection of the two curves. In addition, the small image shows the intersections of *T*_*x*_ and *T*_*y*_ when *W*_*x*_ changes from 50 to 100 nm. It means the ellipticity |*T*_*y*_|/|*T*_*x*_| = 1, so that the proposed structure can realize the conversion of quarter waveplate from LTC polarization. The efficiency is about 0.44, which is close to the ideal transmittance of 0.5 proved by surface admittance method in previous literature [28]. Moreover, when the aperture width *W*_*x*_ is increased from 50 to 100 nm, the operating wavelength is shifted from 1518 (transmittance of about 0.43) to 1550 nm (transmittance of about 0.44). This means the proposed work has a good robustness, and it is beneficial to the experimental preparation.

**Fig. 3:**
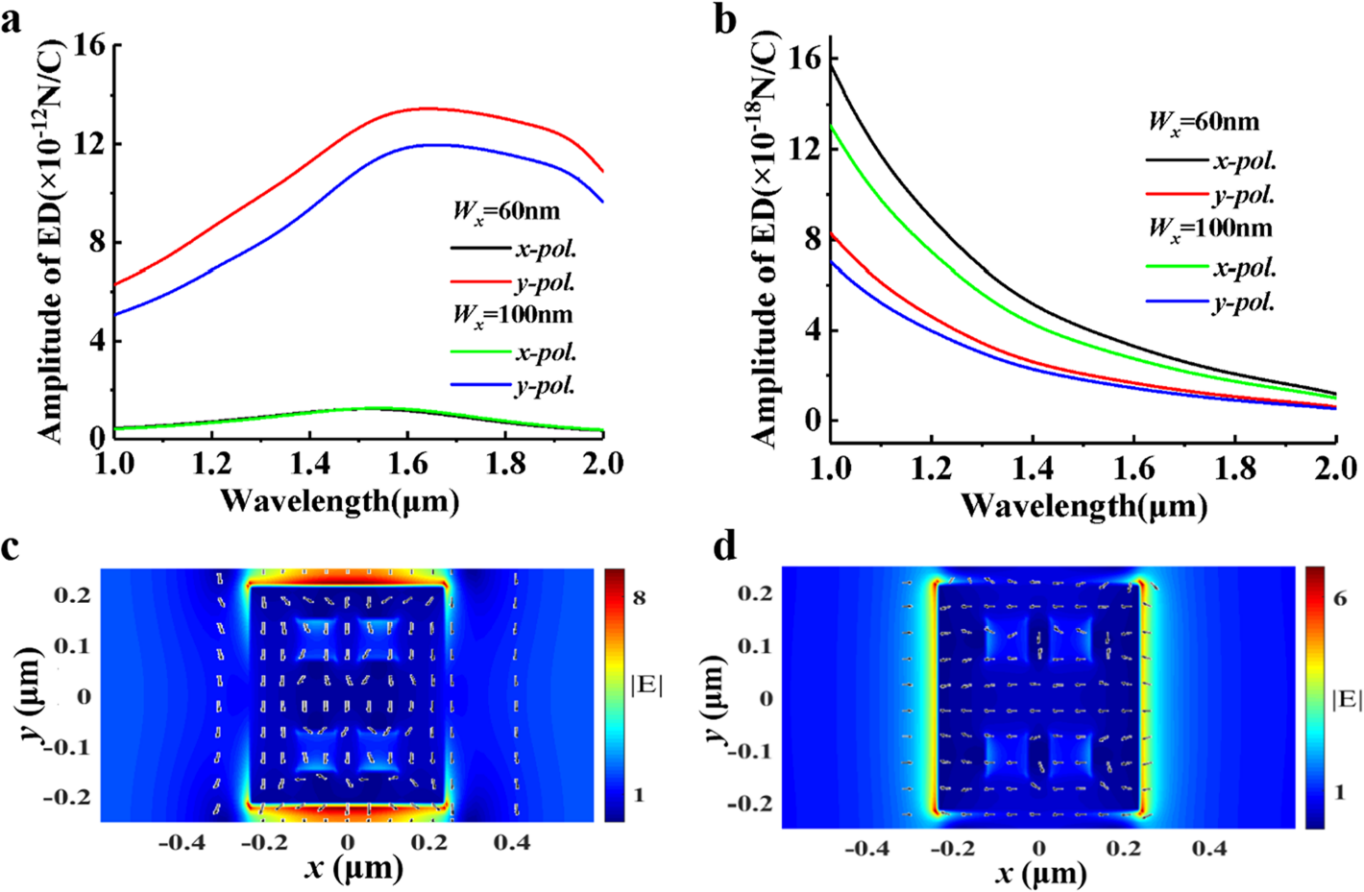
**a** The intensity of ED resonances. **b** The intensity of MD resonances. **c, d** The electric field intensity and vectors of *x-pol.* and *y-pol.* incidence, respectively

We numerically analyze the resonances of electric and magnetic dipoles (ED and MD) under *x-pol.* and *y-pol.* at different *W*_*x*_. It can be seen from Fig. 3a, b that there is hardly any MD resonance in two polarization directions and there exists ED resonance at 1550 nm for *x-*polarization and 1600 nm for *y-*polarization. Figure 3c shows the intensity and direction of the electric field under *x-pol.* incidence (λ = 1550 nm) and Fig. 3d for *y-pol.* (λ = 1600 nm). The ED resonances can be seen from the direction indicated by the vector arrows. The change of *W*_*x*_ has little effect on the dipole resonance of *x-pol.*, but *y-pol.* is relatively affected. Through changing the range of Wood’s anomaly and the position of electric dipole, the transmission, phase, and polarization of our design can be better controlled. This enables us to obtain better quarter waveplate performance in the near infrared band. It also provides a new idea for the design of metasurface waveplate [33–41].

In order to examine the operating band of the quarter waveplates and the performance at the communication wavelength around 1550 nm, we divide the comparisons into four parts (shown in Table 1): circular polarizability at 1550 nm, transmission efficiency at 1550 nm, the thickness and the bandwidth from circular polarization to linear polarization can be achieved.

**Table 1:**
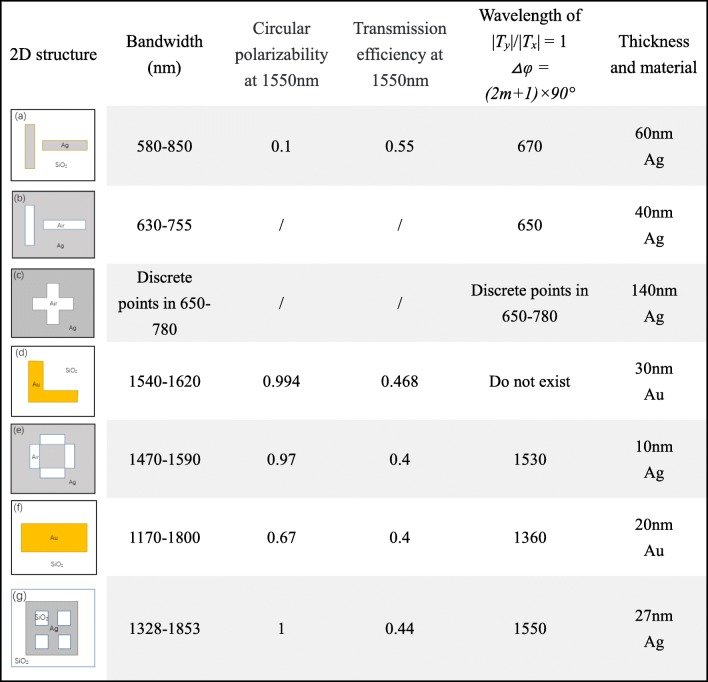
Comparison of the characteristics of quarter waveplates in several papers mentioned above

The first column of Table 1 is the top view (two-dimensional) of the structures, which is only a schematic diagram, and does not show the specific size and proportion. The materials are simply shown in the figures. The second column is the bandwidth of the structure as quarter waveplates, in which circular polarization can be converted to linear polarization, and the phase difference range is 90° ± 5°. The third column is the ellipticity of the LTC polarization transmission at 1550 nm, and the ellipicity |*T*_*y*_|/|*T*_*x*_|. The fourth column is the corresponding wavelength when the ellipticity |*T*_*y*_|/|*T*_*x*_| = 1, and the *△φ = φ*_*y*_*-φ*_*x*_ *= (2 m + 1) × 90°* simultaneously, where *m* is an integer. The fifth column is the thickness of the metal layer of each quarter waveplate and silica is the only other material. The results of all the above articles are from simulations, using FEM, FDTD, and so on.

**Fig. 4:**
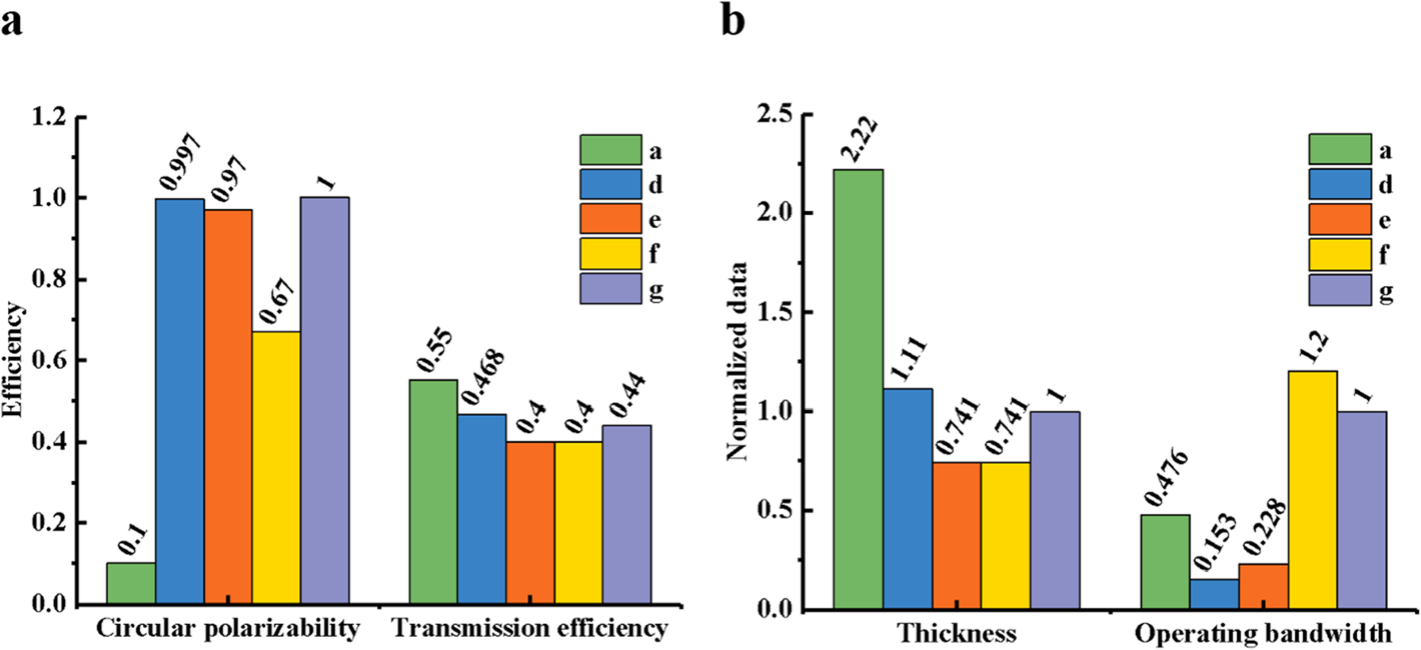
The comparison of the characteristics of the structures mentioned in Table 1a, d, e, f, and g. **a** The ellipticity of LTC polarization and the total transmission at 1550 nm. **b** The normalized metal thickness, and the normalized bandwidth of CTL polarization, based on the proposed structure g

The performance of five structures working at communication bandwidth in Table 1a, d, e, f, and g is presented as bar graphs. They represent the nanorods, L-shaped, broken rectangular annulus arrays, single-layer gold nanorod array, and two by two rectangle-holed silver film structures, respectively. The circular polarizability and transmission efficiency of different quarter waveplates at 1550 nm are shown in Fig. 4a, and their respective metal layer thickness and working bandwidth are shown in Fig. 4b. For convenience, we normalize the thickness and bandwidth, which is based on the metal thickness (27 nm) and the operating bandwidth (525 nm) proposed in this work.

By comparing the five structures mentioned above, we find that, though structure a has the highest transmission efficiency and a wide band, it is completely impossible to achieve circular polarization at 1550 nm, and has a very large thickness. Structure d has the highest circular polarizability, a high transmission efficiency and thickness ranks second in five, but the bandwidth is very narrow. This design can achieve CTL and LTC polarization well at 1550 nm, but it is not suitable for a quarter waveplate with large bandwidth. Ultrathin waveplates e and f have the same thickness of 10 nm, and the same lowest transmission efficiency. However, under the comparison of circular polarizability, e is better than f performance, and bandwidth, f is far more better than e. Although structure f has the widest band, the other three indicators are all the worst, and it is impossible to achieve circular polarization at 1550 nm. Structure g not only realizes the LTC/CTL transformations efficiently and perfectly, but also has the characteristics of small thickness and wide working band. This is the result of weighing the necessary performance of a quarter waveplate. Combining the existing nanoprocessing technology with published literatures, we found that our quarter waveplate can be prepared experimentally. Generally speaking, we can accomplish the experiment in three steps: first, rectangle-shaped patterns are defined on the ZEP520 resist layer by electron beam lithography (EBL) on silica substrate; second, a quarter waveplate supercell complementary structure array is obtained by electron beam exposure; third, a thin silver layer is deposited by electron beam evaporation; last step, remove unwanted materials by a lift-off or etch-back process. Reference [25] used the same procedure to prepare gold nanorod quarter waveplate. The thickness of silver nanorods is 60 nm, and the narrowest width is 20 nm. The depth-to-width is 3, which means it is relatively difficult to manufacture. Reference [16] made a quarter waveplate using the same processes. The thickness of gold film is 35 nm, and the narrowest metal gap is only 10 nm. Although some inevitable thickness inhomogeneity and material losses reduce the resonance strength at shorter wavelengths, the measurements agree well with the simulations. In this work, the thickness of the silver layer of the waveplate is 27 nm, and the narrowest part is 50 nm, the depth-to-width is about 0.5. Moreover, as shown in the small image of Fig. 2c, when the apertures width *W*_*x*_ is increased from 80 to 100 nm, the operating wavelength is shifted from 1545 (transmittance of about 0.432) to 1550 nm (transmittance of about 0.44). It means the structure of the paper has a good robustness and will not greatly affected by the experimental errors.

Therefore, the several aperture structure avoids the idea of introducing anisotropic phase difference by the slender structure (which is difficult to construct) and provides a new direction for the design of quarter waveplate.

## Conclusions

We have numerically considered a realizable broadband transmissive quarter waveplate at communication wavelength, which has a period array of subwavelength holes on a 27-nm-thick silver film. By adjusting the plasmonic resonances, electric dipole resonances, and Wood’s anomalies, it can achieve a wide circular-to-linear polarization band (525 nm) and a high transmission efficiency of 0.44, which is close to the theoretical maximum value of 0.5 calculated by the surface admittance theory. Especially at 1550 nm, the ellipticity is 1, which perfectly realizes the conversion from linear to circular polarization. Through analysis, we believe that this structure can work well as a quarter waveplate for its good robustness. This is expected to be used in miniaturized optical components such as polarization manipulation, optical sensing, and communication functions.

## Data Availability

The datasets generated during and/or analyzed during the current study are available from the corresponding authors on reasonable request.

## Abbreviations

CTL: Circular-to-linear
LTC: Linear-to-circular
FDTD: Finite-difference time-domain
*θ*_*i*_: The angle of the incident wave
*x-pol.*: x-polarization
*y-pol.*: y-polarization
SiO_2_: Silica
Ag: Silver
Au: Gold
